# Impact of national cancer policies on cancer survival trends and socioeconomic inequalities in England, 1996-2013: population based study

**DOI:** 10.1136/bmj.k764

**Published:** 2018-03-14

**Authors:** Aimilia Exarchakou, Bernard Rachet, Aurélien Belot, Camille Maringe, Michel P Coleman

**Affiliations:** Cancer Survival Group, London School of Hygiene and Tropical Medicine, London WC1E 7HT, UK

## Abstract

**Objective:**

To assess the effectiveness of the NHS Cancer Plan (2000) and subsequent national cancer policy initiatives in improving cancer survival and reducing socioeconomic inequalities in survival in England.

**Design:**

Population based cohort study.

**Setting:**

England.

**Population:**

More than 3.5 million registered patients aged 15-99 with a diagnosis of one of the 24 most common primary, malignant, invasive neoplasms between 1996 and 2013.

**Main outcome measures:**

Age standardised net survival estimates by cancer, sex, year, and deprivation group. These estimates were modelled using regression model with splines to explore changes in the cancer survival trends and in the socioeconomic inequalities in survival.

**Results:**

One year net survival improved steadily from 1996 for 26 of 41 sex-cancer combinations studied, and only from 2001 or 2006 for four cancers. Trends in survival accelerated after 2006 for five cancers. The deprivation gap observed for all 41 sex-cancer combinations among patients with a diagnosis in 1996 persisted until 2013. However, the gap slightly decreased for six cancers among men for which one year survival was more than 65% in 1996, and for cervical and uterine cancers, for which survival was more than 75% in 1996. The deprivation gap widened notably for brain tumours in men and for lung cancer in women.

**Conclusions:**

Little evidence was found of a direct impact of national cancer strategies on one year survival, and no evidence for a reduction in socioeconomic inequalities in cancer survival. These findings emphasise that socioeconomic inequalities in survival remain a major public health problem for a healthcare system founded on equity.

## Introduction

Differences in cancer survival between less and more deprived patients have been well documented for most types of cancer and in different geographical settings.^1^ ^2^ ^3^ ^4^ ^5^ ^6^ ^7^ There is evidence for some explanations related to patient, tumour, and healthcare characteristics, but these can only explain part of the differences depending on the cancer type and healthcare system.^8^ ^9^ Cancer survival in England has been improving steadily since the 1970s,^10^ but socioeconomic inequalities in survival persist for most cancers,^11^ despite concerted efforts and investment in the National Health Service.

After the Calman-Hine report in 1995,^12^ the first fully detailed strategy to tackle cancer in England was the NHS Cancer Plan,^13^ introduced in 2000. It set out the government’s plans for investment and reform, aiming at improving prevention, delivery of care (including implementation of multidisciplinary teams), and research. It led to an inflation adjusted increase of 35% in annual expenditure on cancer services between 2001 and 2004. Among the main aims were improving cancer survival to levels comparable with the rest of Europe and reducing socioeconomic inequalities. In 2007, the Cancer Reform Strategy^14^ focused on consolidation of progress made since publication of the NHS Cancer Plan and set out plans for cancer services over the ensuing five years. Again, tackling inequalities and promoting equality in access to cancer services in England were central to the strategy, which also led to the foundation of the National Cancer Equality Initiative in 2008, a multidisciplinary initiative dedicated to this purpose.^15^ In 2008, the National Awareness and Early Diagnosis Initiative (NAEDI) was launched, with the purpose of stimulating action to diagnose cancer earlier and improve cancer outcomes. Some of the key target areas were tackling negative attitudes to cancer and the barriers to seeing a doctor, supporting primary care, and optimising access to diagnostic tests and referral pathways. These initiatives occurred concomitantly with major reorganisation of the NHS and funding pressure on NHS spending (reduction of the health spend as a proportion of the gross domestic product) after publication of a white paper in 2010.^16^

We investigated the effectiveness of the NHS Cancer Plan and subsequent strategies in improving one year survival and reducing socioeconomic inequalities in cancer survival, up to 14 years after the introduction of the plan, in the context of major changes in the NHS since 2010. We focused on one year survival because most inequalities in cancer survival in England arise shortly after diagnosis.^17^ We examined trends in cancer survival and in the deprivation gap in survival for patients receiving a diagnosis in three predefined calendar periods: 1996-2000 (before the cancer plan), 2001-05 (initialisation period), and 2006-13 (implementation period), with follow-up to 2014. This allowed comparison of trends before and after introduction of the NHS Cancer Plan, including an initialisation period to reflect the latency before such an extensive and wide ranging strategy might take effect. We also analysed the changes in survival patterns without fixing the calendar periods a priori, to examine survival trends after the successive cancer policy initiatives but without imposing assumptions on the calendar periods during which those changes might occur.

## Methods

### Data

We extracted data from the population based National Cancer Registry database held by the Office for National Statistics (ONS). The primary source of cancer registration records is a range of healthcare providers, such as hospitals, pathology laboratories, and other services that provide all the information on the cancer diagnoses in a given year. This information is collected and maintained by the National Cancer Registration and Analysis Service in Public Health England, which actively updates the database for up to nine months after the registration year. The vital status of registered patients with cancer (alive, emigrated, dead, not traced) is updated by ONS and the HSCIC (Health and Social Care Information Centre, now known as NHS Digital). The estimated completeness of this dynamic database is 98% at the registration calendar year, but it can reach 100% within five years.^18^ ^19^

We included all young people and adults (age 15-99 years) with a diagnosis of one of the 24 most common primary, malignant (ICD-O (international classification of diseases for oncology) behaviour code 3), invasive neoplasms between 1996 and 2013, with potential follow-up until the end of 2014. These represent about 91% of all cancers diagnosed in England. Tumour site was coded according to ICD-10 (international classification of diseases, 10th revision),^20^ whereas morphology and behaviour were coded according to the international classification of diseases for oncology, second edition (ICD-O-2).^21^ The data owners undertake various cleaning procedures to ensure high quality of the data, but we also apply a standard set of additional checks for cancer survival analysis, aiming to flag or exclude incomplete, ineligible, or incoherent tumour records, as well as second or higher order tumours arising in the same organ as a previous primary cancer.^22^ Overall, these procedures led to exclusion of less than 5% of patients. The analyses included over 3.5 million patients.

### Deprivation

The index of multiple deprivation (IMD 2004)^23^ is an ecological measure of deprivation, with seven distinct domains and a combined measure, assigned to individuals living within a given Lower-layer Super Output Area (LSOA). LSOAs are administrative geographical areas established to improve reporting of small area statistics in England and Wales. Patients with cancer were assigned to one of 32 482 LSOAs in England (mean population 1500) on the basis of their postcode of residence at diagnosis. For our study we used the income domain score, which measures the proportion of the population with low income in a given LSOA. The five deprivation categories were based on the fifths of the national distribution of scores for the 32 482 LSOAs in England and patients with cancer were assigned to the deprivation category of their LSOA (from 1 indicating “least deprived,” or affluent, to 5 indicating “most deprived”).

### Net survival estimation

We estimated one year net survival for each cancer by sex, year of diagnosis (1996 to 2013), and deprivation category. Patients with a diagnosis between 1996 and 2013 had the potential to be followed up for at least one year, so we used the classic cohort approach.

Net survival is the probability of survival if cancer were the only possible cause of death. It is the only survival measure enabling comparisons between populations (ie, between periods and socioeconomic levels) in which mortality hazard from other causes may differ, because this measure does not depend on these hazards. Estimation of net survival requires the comparison of the overall mortality hazard experienced by the patients with cancer to their expected mortality hazard—that is, hazard from other causes of death. This leads to an estimate of the excess mortality hazard (ie, hazard of death due to the cancer of interest), which mathematically is the complement of net survival.^24^ Because the cause of death is not considered as reliable in population based data, the expected mortality hazard of the patients with cancer is estimated in the general population that the patients come from. We therefore built life tables for the England general population by calendar year, sex, age, and deprivation.^25^ ^26^ In the absence of data on recent deaths in the general population, we used the 2011 mortality rates for 2012 and 2013.

We estimated net survival using the consistent non-parametric estimator defined by Pohar-Perme.^27^ This estimator accounts for the informative censoring due to patient factors such as age—that is, when some groups of patients are more likely to be censored because of death from other causes. The estimator is implemented in Stata 14^28^ within the *stns* command.^29^

### Age standardisation

Survival estimates for all ages combined were age standardised with the International Cancer Survival Standard weights.^30^ Age standardisation required to estimate survival in 18 450 unique combinations of cancer (20 in men and 21 in women), sex, year of diagnosis (18 years), deprivation (five categories), and age groups (five groups). In 562 of these combinations it was not possible to estimate survival owing to sparse data. In those cases, we combined the data for adjacent age groups and assigned the pooled survival estimate to both age groups, the corresponding weights for these age groups being also combined. If survival estimates were missing for more than one age group, we report only the unstandardised survival estimate (382 combinations). These issues arose mostly for mesothelioma, thyroid and testicular cancer, Hodgkin lymphoma, and myeloma, which tend to be rare in either very young or very old patients.

### Trends in survival, deprivation gap, and trends in deprivation gap

We used multivariable linear regression to investigate the survival patterns for each cancer and by sex. The outcome was one year age standardised net survival and the predictors were year of diagnosis (representing the trend) and deprivation. The model also included an interaction between year of diagnosis and deprivation, which defined the temporal trend in the deprivation gap: the significance level of this term was set at 0.05. This allowed us to test the statistical significance of the interaction and to decide if there was evidence for a change in the deprivation gap.

A continuous linear effect was considered for the effect of deprivation. We tested a series of linear restricted regression splines with constrained knot location for the effect of year and the interaction term. Knots were fixed at the calendar years 2001 and 2006, to align with the three periods we defined in relation to the NHS Cancer Plan. The final number of knots was determined with an algorithm embedded in the *mvrs* program in STATA.^31^ Starting with the model of maximum complexity, this closed-test algorithm uses a backward elimination to choose the best fitting spline, while the overall type I error is kept at a predefined level (here 5%).

**Fig 1:**
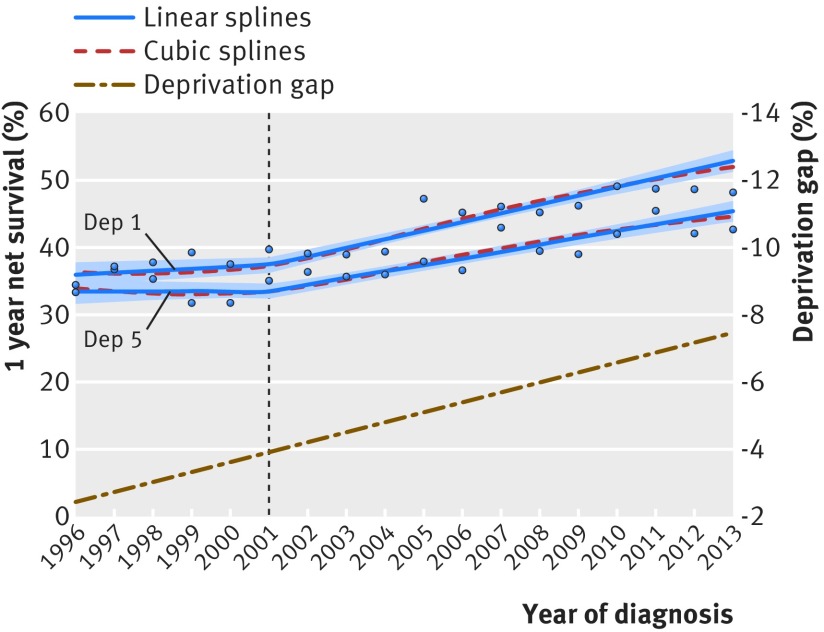
Trends in one year net survival in the least and most deprived, and trends in deprivation gap (absolute difference between least and most deprived categories) for brain cancer in men

From the regression models applied to the entire dataset for each sex-cancer combination we estimated both survival and the deprivation gap in survival for each year. Survival is the predicted age standardised one year net survival for patients with a diagnosis in each calendar year. The deprivation gap is the absolute difference between the predicted net survival estimates for the most affluent and most deprived groups (fig 1). By convention, a negative value for the deprivation gap implies that survival was lower in deprived than in affluent patients. We derived 95% confidence intervals from the linear combination of coefficients acquired from the flexible models.

### Relaxed assumptions

Our main analysis incorporated the assumption that 2001 and 2006 were starting points for any change in the slope of the trend in survival or in the deprivation gap in survival. We then relaxed this assumption by including an internal knot for each year in the initial model, again allowing the knots to be selected by the algorithm embedded in the command.^31^ The deprivation gap was derived from the same final models as described previously. We performed similar analyses using cubic splines to allow for the possibility of non-linear trends.

### Patient involvement

This study is part of the Cancer Survival Group’s commitment to describe and explain inequalities in cancer survival affecting older patients, patients of low socioeconomic status, and all patients living in England and in the UK, where cancer survival still lags behind survival in other comparably wealthy countries.

We repeatedly receive feedback from patients with cancer and advocacy bodies at national and international meetings to the effect that the cancer survival statistics we produce are an invaluable support for their efforts to lobby for improved care of patients with cancer. We have a longstanding collaboration with the National Cancer Research Institute Consumer Liaison Group—a group of patients’ representatives that is actively involved in our research. We organise regular meetings at which we discuss our research, exchange ideas, and receive valuable feedback. More than 40 members of this group participated in our most recent meeting, on 13 February 2017, at which our research (including this study) was presented and discussed in plenary session and in small groups. Two patients are also members of the Advisory Panel for the Cancer Survival Programme, of which this study is a component part. We recently received special recognition from Cancer Research UK for the involvement and engagement of patients in the design and delivery of our research.

Our international research programme on cancer survival is also officially endorsed by many cancer patient bodies, including the Association of European Cancer Leagues (Brussels, Belgium), the European Institute for Women’s Health (Dublin, Ireland), and the European Cancer Patient Coalition. These agencies have all used our cancer survival estimates to press for improvements in cancer care locally, but also to improve cancer policy nationally.

## Results

### Trends in one year net survival

One year survival improved for 20 of the 21 cancers examined in women and 16 of the 20 cancers examined in men (table 1).

**Table 1:** Age standardised one year net survival (%) in men and women with a diagnosis of one of 24 cancers in 1996, and mean annual change (%) in successive calendar periods 1996-2013, England

Malignancy	Men					Women			
	Survival* 1996 (95% CI)	Mean annual change (95% CI)				Survival* 1996 (95% CI)	Mean annual change (95% CI)		
		1996-2000	2001-05	2006-13			1996-2000	2001-05	2006-13
Oesophagus	29.4 (28.1 to 30.7)	← 1.1 (1.0 to 1.2) →				31.7 (30.0 to 33.3)	← 1.0 (0.8 to 1.1) →		
Stomach	34.9 (33.7 to 36.1)	← 0.9 (0.7 to 1.0) →				35.9 (34.5 to 37.3)	← 0.7 (0.6 to 0.8) →		
Colon	67.0 (65.9 to 68.2)	← 0.7 (0.5 to 0.8) →				78.7 (77.6 to 79.8)	← 0.6 (0.4 to 0.7) →		
Rectum	73.2 (71.9 to 74.5)	← 0.6 (0.5 to 0.7) →				74.3 (73.1 to 75.4)	← 0.5 (0.4 to 0.6) →		
Liver	18.7 (17.3 to 20.1)	← 1.1 (0.9 to 1.2) →				21.1 (19.1 to 23.0)	← 0.7 (0.5 to 0.9) →		
Pancreas	13.2 (12.0 to 14.4)	← 0.7 (0.5 to 0.8) →				13.4 (12.3 to 14.5)	← 0.9 (0.7 to 1.0) →		
Larynx	82.7 (81.4 to 84.0)	← 0.1 (0.0 to 0.3) →							
Lung	24.0 (23.0 to 24.9)	← 0.5 (0.4 to 0.7) →		1.0 (0.8 to 1.3)		25.4 (24.5 to 26.4)	← 0.8 (0.6 to 0.9) →		
Mesothelioma	28.3 (26.3 to 30.3)	0.2 (-0.3 to 0.8)	← 1.3 (1.1 to 1.5) →			28.4 (26.0 to 30.8)	← 1.1 (0.8 to 1.3) →		
Melanoma	92.2 (91.4 to 93.0)	← 0.3 (0.2 to 0.3) →				95.6 (95.2 to 96.0)	← 0.2 (0.1 to 0.2) →		
Breast						90.1 (89.5 to 90.6)	← 0.4 (0.3 to 0.4) →		
Cervix						78.7 (77.6 to 79.8)	← 0.2 (0.1 to 0.3) →		
Uterus						83.8 (83.0 to 84.6)	← 0.4 (0.3 to 0.5) →		
Ovary						58.4 (57.2 to 59.5)	← 0.8 (0.7 to 1.0) →		
Prostate	81.3 (80.4 to 82.2)	1.2 (0.9 to 1.5)	0.1 (-0.1 to 0.3)	0.6 (0.5 to 0.8)					
Testis	95.9 (84.7 to 97.2)	← 0.0 (-0.1 to 0.2) →							
Bladder	82.9 (81.4 to 84.5)	-0.9 (-1.4 to -0.5)	← 0.1 (0.0 to 0.3) →			75.1 (72.6 to 77.6)	-1.4 (-2.1 to 0.7)	← -0.1 (-0.3 to 0.2) →	
Kidney	60.9 (59.4 to 62.4)	← 0.6 (0.4 to 0.9) →		1.4 (1.1 to 1.8)		59.3 (57.8 to 60.8)	← 0.8 (0.6 to 1.0) →		1.5 (1.2 to 1.9)
Brain	34.7 (32.9 to 36.4)	0.2 (-0.3 to 0.6)	← 1.1 (1.0 to 1.3) →			33.7 (32.4 to 34.9)	← 0.9 (0.8 to 1.0) →		
Thyroid	83.1 (81.1 to 85.1)	← 0.0 (-0.3 to 0.4) →		1.1 (0.6 to 1.5)		83.1 (81.8 to 84.3)	← 0.7 (0.5 to 0.8) →		
Non-Hodgkin lymphoma	63.9 (62.6 to 65.1)	← 0.9 (0.8 to 1.0) →				66.9 (65.7 to 68.0)	← 0.9 (0.8 to 1.0) →		
Hodgkin lymphoma	87.5 (86.3 to 88.7)	← 0.1 (-0.1 to 0.2) →				89.1 (87.6 to 90.5)	← 0.0 (-0.3 to 0.2) →		0.5 (0.1 to 0.8)
Myeloma	63.1 (61.6 to 64.6)	← 0.8 (0.5 to 1.0) →		1.9 (1.6 to 2.2)		62.3 (61.1 to 63.4)	← 1.1 (1.0 to 1.2) →		
Leukaemia	62.4 (61.1 to 63.7)	← 0.3 (0.1 to 0.5) →		1.0 (0.7 to 1.3)		59.2 (57.9 to 60.5)	← 0.6 (0.4 to 0.7) →		

* Derived from the best fitting linear regression model for each cancer.

**Fig 2:**
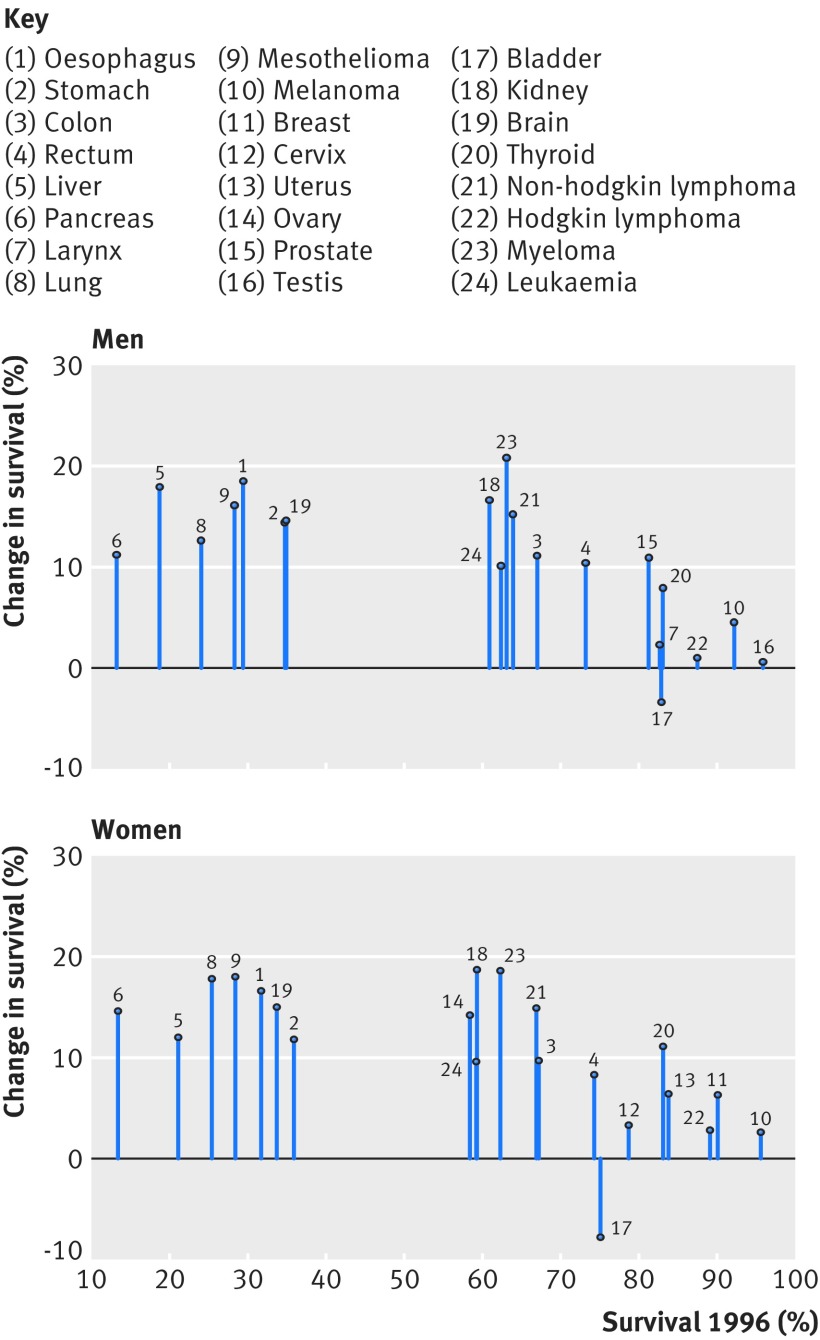
Change in one year net survival between 1996 and 2013 for 20 cancers in men and 21 cancers in women, arrayed by ICD-10

The largest improvements were observed for cancers that were of poor or intermediate prognosis in the 1990s (<65% for those with a diagnosis in 1996), such as cancers of the oesophagus, liver (men), lung (women), and kidney, mesothelioma, and myeloma. For these cancers, the average annual absolute increase in one year age standardised net survival was often greater than 1% over the whole study period (fig 2). Survival for men diagnosed as having cancer of the larynx or testis, or Hodgkin lymphoma, was already high in the 1990s, and it improved little by 2013.

For 26 of the 41 cancer-sex combinations, survival improved steadily from 1996, but with no statistically significant acceleration after 2006, ie, after the predefined implementation period. This was the case for eight of the 20 malignancies in men: six cancers of the digestive tract, melanoma, and non-Hodgkin lymphoma; and for 18 of 21 malignancies in women: six cancers of the digestive tract, lung cancer, mesothelioma, melanoma, four gynaecological cancers, brain cancer, thyroid cancer, non-Hodgkin lymphoma, myeloma, and leukaemia.

Changes in the survival trend were observed for several cancers. For mesothelioma in men, one year survival changed little during 1996-2000 (mean annual increase 0.2%), but accelerated to 1.3% each year during 2001-13 (table 1). A similar change occurred for brain tumours in men at the same time point (0.2% to 1.1% each year).

For thyroid cancer in men, one year survival changed little during the 10 year period 1996-2005, but then increased by 1.1% each year between 2006 and 2013. A similar pattern was seen for Hodgkin lymphoma in women, which increased by 0.5% a year between 2006 and 2013.

The one year survival trends seen during 1996-2005 accelerated from 2006 for lung cancer, myeloma, and leukaemia in men, and for kidney cancer in both sexes. The average annual increases during 1996-2005 were less than 1% a year, but increased up to 2% a year between 2006 and 2013. For kidney cancer, the annual rate of increase in one year survival doubled from 2006, increasing from 0.6% to 1.4% a year in men, and from 0.8% to 1.5% a year in women.

For prostate cancer, the mean annual increase in one year survival was 1.2% during 1996-2000, null during 2001-05, and 0.6% during 2006-13; by 2013, one year survival had reached 92.1%.

When we relaxed the assumption that the trend could only change in 2001 or 2006, fitting flexible splines that allow the trend to change from year to year, the results differed little (data not shown).

### Deprivation gap in one year net survival and trends

**Fig 3:**
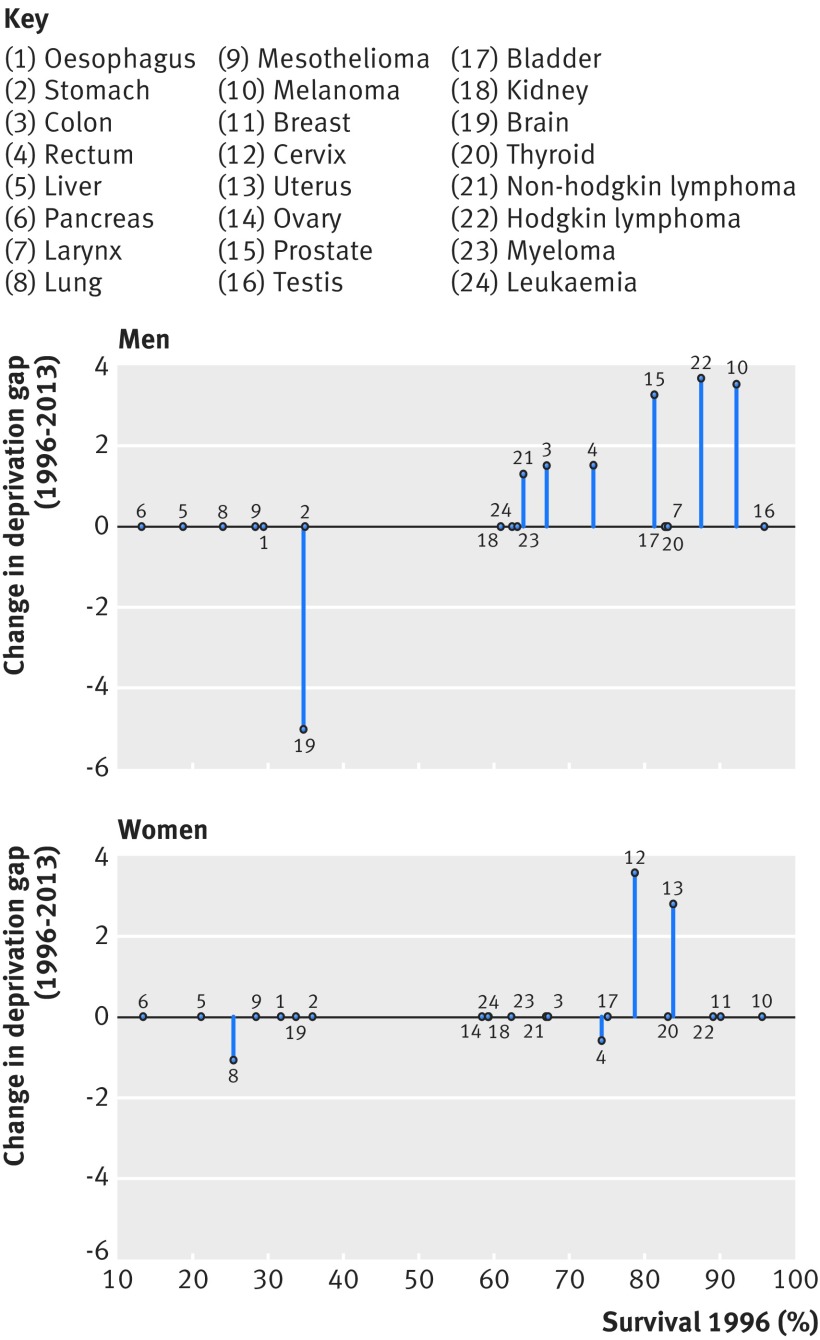
Change in deprivation gap in one year net survival between 1996 and 2013 for 20 cancers in men and 21 cancers in women, arrayed by ICD-10

When survival increased, it concerned all deprivation groups for most sex-cancer combinations. Survival nevertheless remained consistently lower among more deprived patients than the less deprived, and the deprivation gap in one year net survival remained unchanged for 13 cancers in men and 17 cancers in women between 1996 and 2013 (fig 3). The survival gap narrowed only in six out of 20 cancers among men and in two out of 21 cancers among women, and widened for three cancers. All these changes were linear. The deprivation gaps were more similar between men and women in 2013 than in 1996.

In 1996 there was a clear deprivation gradient in one year survival, which was lower among more deprived than less deprived patients, for all cancers and in both sexes (tables 2 and 3). Seventeen years later, in 2013, survival was still lower among the more deprived groups for all cancers, except Hodgkin lymphoma in men. A narrowing in the deprivation gap was observed for cancers with survival in 1996 near or higher than 65% among men and 75% among women.

**Table 2:** Adjusted one year survival and change in net survival for men with a diagnosis of one of 20 cancers between 1996 and 2013

Malignancy	1996			2001			2006				2013			1996-2013
	Survival in most affluent (95% CI)	Deprivation gap (95% CI)		Survival in most affluent (95% CI)	Deprivation gap (95% CI)		Survival in most affluent (95% CI)		Deprivation gap (95% CI)		Survival in most affluent (95% CI)	Deprivation gap (95% CI)		Change in deprivation gap (%)
Oesophagus	32.4 (31.4 to 33.3)	−7.3 (−8.4 to −6.3)		38.6 (37.9 to 39.3)	−7.3 (−8.4 to −6.3)		44.8 (44.0 to 45.7)		−7.3 (−8.4 to −6.3)		50.6 (49.5 to 51.7)	−7.3 (−8.4 to −6.3)		0.0
Stomach	37.8 (36.8 to 38.9)	−5.8 (−7.1 to −4.6)		42.1 (41.3 to 42.9)	−5.8 (−7.1 to −4.6)		46.4 (45.6 to 47.1)		−5.8 (−7.1 to −4.6)		52.4 (51.3 to 53.4)	−5.8 (−7.1 to −4.6)		0.0
Colon	71.0 (70.1 to 72.0)	−7.9 (−9.2 to −6.5)		73.5 (72.9 to 74.1)	−7.4 (−8.3 to −6.5)		76.0 (75.3 to 76.7)		−7.0 (−7.7 to −6.2)		81.6 (80.6 to 82.5)	−6.3 (−7.7 to −5.0)		1.6
Rectum	77.9 (77.0 to 78.8)	−9.4 (−10.7 to −8.0)		80.0 (79.5 to 80.6)	−8.9 (−9.8 to −8.0)		82.2 (81.5 to 82.8)		−8.5 (−9.2 to −7.7)		87.7 (86.8 to 88.7)	−7.8 (−9.2 to −6.5)		1.6
Liver	21.5 (20.1 to 22.9)	−5.7 (−7.4 to −3.9)		26.8 (25.6 to 27.9)	−5.7 (−7.4 to −3.9)		32.0 (31.0 to 33.1)		−5.7 (−7.4 to −3.9)		39.4 (38.0 to 40.8)	−5.7 (−7.4 to −3.9)		0.0
Pancreas	16.2 (15.1 to 17.2)	−5.9 (−7.1 to −4.7)		19.5 (18.7 to 20.3)	−5.9 (−7.1 to −4.7)		22.8 (22.0 to 23.5)		−5.9 (−7.1 to −4.7)		27.4 (26.3 to 28.4)	−5.9 (−7.1 to −4.7)		0.0
Larynx	85.9 (84.7 to 87.1)	−6.3 (−7.8 to −4.9)		86.5 (85.6 to 87.5)	−6.3 (−7.8 to −4.9)		87.2 (86.4 to 88.1)		−6.3 (−7.8 to −4.9)		88.2 (87.0 to 89.4)	−6.3 (−7.8 to −4.9)		0.0
Lung	25.9 (25.2 to 26.7)	−4.0 (−4.7 to −3.2)		28.6 (28.1 to 29.1)	−4.0 (−4.7 to −3.2)		31.3 (30.6 to 32.0)		−4.0 (−4.7 to −3.2)		38.6 (37.7 to 39.4)	−4.0 (−4.7 to −3.2)		0.0
Mesothelioma	29.7 (27.6 to 31.9)	−2.9 (−4.7 to −1.0)		30.9 (29.3 to 32.5)	−2.9 (−4.7 to −1.0)		37.1 (36 to 38.3)		−2.9 (−4.7 to −1.0)		45.9 (44.2 to 47.6)	−2.9 (−4.7 to −1.0)		0.0
Melanoma	94.8 (93.9 to 95.6)	−6.2 (−7.6 to −4.8)		95.6 (95.0 to 96.1)	−5.2 (−6.0 to −4.3)		96.4 (95.9 to 96.9)		−4.1 (−4.9 to −3.4)		97.5 (96.6 to 98.3)	−2.7 (−4.1 to −1.3)		3.5
Prostate	83.6 (82.8 to 84.4)	−4.6 (−5.7 to −3.6)		89.0 (88.5 to 89.6)	−3.7 (−4.3 to −3.0)		89.1 (88.6 to 89.6)		−2.7 (−3.3 to −2.1)		92.8 (92.1 to 93.5)	−1.4 (−2.4 to −0.3)		3.2
Testis	97.3 (95.9 to 98.8)	−2.8 (−4.5 to −1.1)		97.5 (96.4 to 98.7)	−2.8 (−4.5 to −1.1)		97.7 (96.7 to 98.8)	−2.8 (−4.5 to −1.1)			98.0 (96.5 to 99.4)	−2.8 (−4.5 to −1.1)		0.0
			
Bladder	85.6 (84.6 to 86.6)	−5.8 (−6.6 to −4.9)		81.7 (80.9 to 82.5)	−5.8 (−6.6 to −4.9)		80.9 (80.1 to 81.7)	−5.8 (−6.6 to −4.9)			83.0 (82.1 to 83.9)	−5.8 (−6.6 to −4.9)		0.0
Kidney	64.1 (62.9 to 65.3)	−6.3 (−7.6 to −5.1)		67.3 (66.5 to 68.1)	−6.3 (−7.6 to −5.1)		70.5 (69.5 to 71.6)	−6.3 (−7.6 to −5.1)			80.7 (79.4 to 82.0)	−6.3 (−7.6 to −5.1)		0.0
Brain	35.9 (34.1 to 37.7)	−2.4 (−4.9 to 0.1)		37.4 (36.2 to 38.6)	−3.9 (−5.5 to −2.3)		43.8 (43.0 to 44.7)	−5.4 (−6.7 to −4.0)			52.8 (51.2 to 54.4)	−7.5 (−10.0 to −5.0)		−5.1
Thyroid	84.4 (82.1 to 86.7)	−2.7 (−5.1 to −0.3)		84.6 (83.1 to 86.2)	−2.7 (−5.1 to −0.3)		84.9 (82.8 to 86.9)	−2.7 (−5.1 to −0.3)			92.3 (89.8 to 94.8)	−2.7 (−5.1 to −0.3)		0.0
Non-Hodgkin lymphoma	67.4 (66.2 to 68.5)	−8.2 (−9.9 to −6.4)		71.6 (70.9 to 72.4)	−7.8 (−8.9 to −6.7)		75.9 (75.2 to 76.6)	−7.4 (−8.4 to −6.5)			81.9 (80.8 to 83.0)	−6.9 (−8.6 to −5.1)		1.3
Hodgkin lymphoma	90.4 (88.3 to 92.5)	−5.1 (−8.3 to −2.0)		89.0 (87.8 to 90.3)	−4.0 (−6.1 to −2.0)		87.6 (86.2 to 89.1)	−3.0 (−4.7 to −1.2)			90.0 (87.8 to 92.1)	−1.4 (−4.6 to 1.7)		3.7
Myeloma	65.9 (64.6 to 67.2)	−5.6 (−7.0 to −4.3)		69.7 (68.8 to 70.6)	−5.6 (−7.0 to −4.3)		73.4 (72.3 to 74.6)	−5.6 (−7.0 to −4.3)			86.7 (85.3 to 88.2)	−5.6 (−7.0 to −4.3)		0.0
Leukaemia	65.1 (64.0 to 66.2)	−5.3 (−6.4 to −4.2)		66.6 (65.9 to 67.4)	−5.3 (−6.4 to −4.2)		68.2 (67.2 to 69.1)	−5.3 (−6.4 to −4.2)			75.2 (74.0 to 76.4)	−5.3 (−6.4 to −4.2)		0.0

**Table 3:** Adjusted one year survival and change in net survival for women with a diagnosis of one of 21 cancers between 1996 and 2013

Malignancy	1996			2001			2006			2013			1996-2013
	Survival in most affluent (95% CI)	Deprivation gap (95% CI)		Survival in most affluent (95% CI)	Deprivation gap (95% CI)		Survival in most affluent (95% CI)	Deprivation gap (95% CI)		Survival in most affluent (95% CI)	Deprivation gap (95% CI)		Change in deprivation gap (%)
Oesophagus	35.7 (34.2 to 37.2)	−8.0 (−9.9 to −6.2)		40.6 (39.4 to 41.8)	−8.0 (−9.9 to −6.2)		45.5 (44.4 to 46.6)	−8.0 (−9.9 to −6.2)		52.3 (50.8 to 53.8)	−8.0 (−9.9 to −6.2)		0.0
Stomach	38.2 (36.7 to 39.7)	−4.6 (−6.4 to −2.8)		41.7 (40.5 to 42.9)	−4.6 (−6.4 to −2.8)		45.2 (44.1 to 46.3)	−4.6 (−6.4 to −2.8)		50.0 (48.5 to 51.6)	−4.6 (−6.4 to −2.8)		0.0
Colon	70.9 (70.2 to 71.6)	−7.4 (−8.3 to −6.5)		73.7 (73.2 to 74.3)	−7.4 (−8.3 to −6.5)		76.6 (76.1 to 77.1)	−7.4 (−8.3 to −6.5)		80.6 (79.9 to 81.3)	−7.4 (−8.3 to −6.5)		0.0
Rectum	75.8 (74.4 to 77.2)	−5.9 (−7.5 to −4.3)		80.0 (79.0 to 81.0)	−6.1 (−7.1 to −5.0)		80.5 (79.7 to 81.4)	−6.3 (−7.1 to −5.4)		86.8 (85.7 to 87.9)	−6.5 (−8.1 to −4.9)		−0.6
Liver	23.7 (21.5 to 26.0)	−5.4 (−8.1 to −2.6)		27.3 (25.5 to 29.0)	−5.4 (−8.1 to −2.6)		30.8 (29.1 to 32.5)	−5.4 (−8.1 to −2.6)		35.7 (33.4 to 38.0)	−5.4 (−8.1 to −2.6)		0.0
Pancreas	16.3 (15.4 to 17.2)	−5.8 (−6.9 to −4.8)		20.6 (19.9 to 21.3)	−5.8 (−6.9 to −4.8)		24.9 (24.2 to 25.5)	−5.8 (−6.9 to −4.8)		30.9 (30.0 to 31.8)	−5.8 (−6.9 to −4.8)		0.0
Lung	27.2 (26.1 to 28.3)	−3.7 (−5.1 to −2.4)		32.5 (31.7 to 33.3)	−4.0 (−4.9 to −3.2)		35.0 (34.3 to 35.7)	−4.3 (−5.1 to −3.6)		45.8 (44.9 to 46.7)	−4.8 (−6.1 to −3.5)		−1.1
Mesothelioma	32.6 (29.9 to 35.2)	−8.3 (−11.5 to −5.1)		37.9 (35.8 to 39.9)	−8.3 (−11.5 to −5.1)		43.2 (41.2 to 45.1)	−8.3 (−11.5 to −5.1)		50.6 (47.9 to 53.2)	−8.3 (−11.5 to −5.1)		0.0
Melanoma	96.9 (96.5 to 97.3)	−1.9 (−2.3 to −1.6)		97.3 (97.0 to 97.5)	−1.9 (−2.3 to −1.6)		97.7 (97.4 to 98.0)	−1.9 (−2.3 to −1.6)		99.6 (99.2 to 100)	−1.9 (−2.3 to −1.6)		0.0
Breast	91.1 (90.7 to 91.5)	−3.2 (−3.6 to −2.8)		93.9 (93.6 to 94.2)	−3.2 (−3.6 to −2.8)		95.5 (95.2 to 95.7)	−3.2 (−3.6 to −2.8)		97.7 (97.4 to 98.1)	−3.2 (−3.6 to −2.8)		0.0
Cervix	84.9 (83.0 to 86.8)	−7.0 (−9.3 to −4.7)		82.8 (81.5 to 84.0)	−6.0 (−7.4 to −4.5)		83.4 (82.5 to 84.3)	−4.9 (−6.2 to −3.7)		84.3 (82.9 to 85.7)	−3.5 (−5.7 to −1.2)		3.5
Uterus	86.2 (85.3 to 87.1)	−5.8 (−7.2 to −4.4)		87.7 (87.1 to 88.2)	−5.0 (−5.9 to −4.1)		89.1 (88.6 to 89.6)	−4.2 (−4.9 to −3.4)		91.2 (90.3 to 92.0)	−3.0 (−4.4 to −1.6)		2.8
Ovary	62.5 (61.6 to 63.4)	−6.8 (−7.7 to −5.8)		65.8 (65.2 to 66.4)	−6.8 (−7.7 to −5.8)		69.1 (68.3 to 70.0)	−6.8 (−7.7 to −5.8)		76.9 (75.9 to 77.9)	−6.8 (−7.7 to −5.8)		0.0
Bladder	79.4 (77.6 to 81.2)	−8.6 (−10.2 to −7.1)		72.5 (71.1 to 73.8)	−8.6 (−10.2 to −7.1)		72.1 (71.1 to 73.1)	−8.6 (−10.2 to −7.1)		71.6 (70.1 to 73)	−8.6 (−10.2 to −7.1)		0.0
Kidney	61.7 (60.2 to 63.1)	−4.7 (−6.2 to −3.1)		65.6 (64.6 to 66.6)	−4.7 (−6.2 to −3.1)		69.5 (68.2 to 70.8)	−4.7 (−6.2 to −3.1)		80.3 (78.7 to 81.9)	−4.7 (−6.2 to −3.1)		0.0
Brain	35.8 (34.4 to 37.1)	−4.2 (−5.9 to −2.6)		40.2 (39.1 to 41.2)	−4.2 (−5.9 to −2.6)		44.6 (43.6 to 45.6)	−4.2 (−5.9 to −2.6)		50.8 (49.4 to 52.1)	−4.2 (−5.9 to −2.6)		0.0
Thyroid	84.4 (82.9 to 85.9)	−2.6 (−4.4 to −0.9)		87.7 (86.5 to 88.8)	−2.6 (−4.4 to −0.9)		90.9 (89.9 to 92)	−2.6 (−4.4 to −0.9)		95.5 (94.1 to 97.0)	−2.6 (−4.4 to −0.9)		0.0
Non-Hodgkin lymphoma	70.4 (69.7 to 71.2)	−7.1 (−8.0 to −6.2)		74.8 (74.2 to 75.4)	−7.1 (−8.0 to −6.2)		79.2 (78.6 to 79.7)	−7.1 (−8.0 to −6.2)		85.3 (84.6 to 86.1)	−7.1 (−8.0 to −6.2)		0.0
Hodgkin lymphoma	88.9 (86.9 to 90.8)	−1.9 (−3.6 to −0.2)		91.1 (89.4 to 92.8)	−1.9 (−3.6 to −0.2)		88.9 (87.3 to 90.5)	−1.9 (−3.6 to −0.2)		93.0 (91.3 to 94.8)	−1.9 (−3.6 to −0.2)		0.0
Myeloma	63.9 (62.6 to 65.2)	−3.4 (−4.9 to −1.8)		69.4 (68.4 to 70.4)	−3.4 (−4.9 to −1.8)		74.9 (73.9 to 75.9)	−3.4 (−4.9 to −1.8)		82.6 (81.3 to 83.9)	−3.4 (−4.9 to −1.8)		0.0
Leukaemia	62.4 (61.2 to 63.6)	−6.5 (−7.9 to −5.0)		65.3 (64.3 to 66.2)	−6.5 (−7.9 to −5.0)		68.1 (67.2 to 69.0)	−6.5 (−7.9 to −5.0)		72.1 (70.9 to 73.3)	−6.5 (−7.9 to −5.0)		0.0

In 1996, the largest deprivation gap in men was observed for rectal cancer (−9.4%) and non-Hodgkin lymphoma (−8.2%). The deprivation gap narrowed slightly by 1.6% during 1996-2013 for both colon and rectal cancer, and by 1.3% for non-Hodgkin lymphoma. However, the largest reduction was seen for Hodgkin lymphoma (3.7%) and prostate cancer (3.2%). For melanoma of the skin, the deprivation gap decreased by 3.5% between 1996 and 2013. The deprivation gap for these cancers ranged from −6.2% to −4.6% in 1996. In 2013, the largest deprivation gap was for rectal cancer (−7.8%) and brain cancer (−7.5%).

In women, the largest deprivation gap in 1996, as in 2013, was for bladder cancer (−8.6%), mesothelioma (−8.3%), and oesophageal cancer (−8%). A reduction was only seen for cervical cancer (from −7.0% in 1996 to −3.5% in 2013) and uterine cancer (from −5.8% to −2.8%, respectively).

The deprivation gap in survival widened for brain tumours in men and lung cancer in women, by 5.1% (from −2.4% in 1996 to-7.5% in 2013) and 1.1% (from −3.7% in 1996 to −4.8% in 2013), respectively.

The deprivation gap was narrow in 1996 for a few malignancies and remained among the narrowest in 2013: Hodgkin lymphoma (−1.9%) and skin melanoma (−1.9%) in women, and thyroid (−2.7%) and testicular cancers (−2.8%) in men.

## Discussion

A steady improvement in one year net survival was seen between 1996 and 2013 in England for nearly all 41 cancer-sex combinations. In 2013, one year net survival was higher than 80% for 17 cancer-sex combinations, but this encouraging picture is moderated by the 14 poor prognosis combinations with one year survival still below 50%. Acceleration of this overall improvement was rarely observed, offering little evidence for a direct impact of the NHS Cancer Plan (2000) and later policy initiatives on short term cancer survival. Meanwhile, the deprivation gap in one year net survival remained unchanged for most cancers, with a clear, persistent pattern of lower survival among more deprived patients. Reduction of socioeconomic inequalities was seen only among some cancers for which one year survival was already more than 65% in 1996, especially among men, suggesting a ceiling effect in that survival has reached a maximum among the least deprived patients.

The successive national policy initiatives, including the 2000 Cancer Plan for England, aimed to improve cancer survival, with the target of bringing survival to the level of comparably wealthy countries, and to reduce the inequalities in cancer survival. The lack of consistent results between men and women, as well as the lack of general patterns across cancer types, provide little evidence for any strong impact of the national cancer policies on short term cancer survival. The evidence is even weaker for their impact on the socioeconomic inequalities in cancer survival.

### Strengths and weaknesses of this study

A major strength of this study is that it is based on virtually all cancer cases registered in England, and the quality and completeness of the English cancer registry data are acknowledged to be high.^32^ The study also updates by seven years our previous evaluations,^11^ ^17^ ^33^ with a total of 18 years of incidence data. These extra years of data allowed us to estimate the trends more accurately.

Since our previous evaluations new, more flexible methodologies were introduced. The assumption that trends in survival and in deprivation gap should be different in three predefined periods^11^ was now relaxed and the periods could vary substantially. The initial assumption was that changes would be expected after 2001 or 2006, or both but further analyses were conducted using more flexible models, which enabled the number and location of the knots to vary across all years of diagnosis. The estimates were not all identical, but they did not affect our main conclusions, in particular on the common absence of inflexion points in the trends in survival and in deprivation gap.

Short term net survival mostly reflects the speed of patient management (including diagnosis, staging, and first definitive treatment) as well as the quality of the surgical treatment and postoperative care. A persistent deficit in short term cancer survival in England (and more generally in the UK) compared with most wealthy countries has been observed for decades.^34^ ^35^ Meanwhile, the wide socioeconomic inequalities in cancer survival, also seen for decades, are mostly due to higher short term mortality in more deprived patients.^4^

Although trends in cancer survival have been regularly used to inform governments on the progress towards the aims of their cancer policies,^36^ ^37^ to our knowledge, little has been specifically published on the evaluation of how cancer policies impact survival and inequalities at national level. Most studies were at subnational level 38 or focused on very specific interventions, such as screening.^39^ By contrast, our study was designed to evaluate such policies. We acknowledge that changes in the survival trends are decided solely on acceleration in survival, and comparison with countries of similar wealth would put any observed improvements in perspective. This limitation, however, does not apply to our findings on the persistent socioeconomic inequalities in cancer survival. Furthermore, the weak evidence for an acceleration in cancer survival echoes the constant gap in cancer survival between England and some other wealthy countries.^40^ Our study also may be too early to detect the full impact of the recently implemented cancer initiatives, although it confirms the findings of our earlier studies.^11^ ^33^ Such studies should be regularly updated.

### Meaning of the study

Since the introduction of the NHS Cancer Plan (2000), acceleration in the positive survival trends was witnessed only for a few cancers and mostly among men, who experienced a lower initial increase compared with women (cancer of the lung, brain, and thyroid, mesothelioma, myeloma, and leukaemia). No such acceleration was found among women. For lung cancer, and more specifically non-small cell carcinoma, the proportion of patients receiving a surgical treatment was low in England,^41^ but this proportion increased from around 10% until 2008^42^ to 17% in 2015.^43^ This improvement may be partly the result of a higher number of specialised surgeons^44^ and a higher proportion of patients managed in specialised centres, which could reduce the variability in postoperative mortality.^45^ These changes may have impacted the outcome for mesothelioma, too. The continuous expansion in the availability of diagnostic tools (eg, computed tomography, magnetic resonance imaging, ultrasound machines) in England is likely to have increased the proportion of brain and thyroid tumours diagnosed at an earlier stage.^46^ Survival pattern for bladder cancer is particular as one year survival decreased slightly between 1996 and 2001, then stabilised. It reflects a change in coding around 2000, under which papillomas were reclassified from invasive to uncertain (whether benign or malignant), therefore excluded from survival analyses. Omitting these tumours with a good prognosis resulted in a decrease in cancer survival.^47^ Despite these improvements in survival there was no reduction in the inequalities in survival from lung, brain, or thyroid tumour, or from mesothelioma.

Particular efforts were dedicated in England to high incidence cancers with intermediate prognosis (one year survival between 40% and 65% in 1996) such as colon and rectal cancers, and one could have expected a faster improvement in survival and a reduction of the deprivation gap after the policy initiatives. Survival from these cancers in England remained behind internationally,^40^ ^48^ and inequalities in survival from these cancers hardly narrowed. Short term survival increased dramatically since 1996 for most other digestive cancers with poor prognosis (one year survival <40% in 1996), but the more deprived patients still experienced lower survival.

It is likely that the longstanding deficit in survival and the socioeconomic inequalities in survival in England share the same causal factors, which can be grouped into patient, tumour, and healthcare system factors. The National Awareness and Early Diagnosis Initiative^49^ and the Be Clear on Cancer Campaign^50^ aimed specifically to tackle some of the patient related (cancer awareness, barriers) and tumour related (tumour stage) issues. Although cancer awareness varies internationally^51^ and by deprivation,^52^ it seems to explain none of the international disparities in cancer survival^51^ and little of socioeconomic inequalities.^53^ A lot of effort has also gone into diagnosing cancers at an earlier stage. Patients tend to have a diagnosis of more advanced tumours in England compared with wealthy countries,^54^ ^55^ ^56^ ^57^ and among the more deprived patients compared with the least deprived.^9^ However, as stage specific survival tends to be lower in England, more advanced stage would explain only part of the international^54^ ^55^ ^56^ ^57^ and socioeconomic inequalities in cancer survival.^58^ ^59^ A higher proportion of patients are now receiving a diagnosis through Two Week Wait or GP referral while for some cancers there is a major decrease in emergency presentation.^60^ Although stage distribution might have slightly moved towards earlier stages, the picture remains patchy and there was no evidence to suggest a narrowing of these gaps in survival.

These policy initiatives put a greater emphasis on individual factors than on the observed suboptimal management of patients with cancer. The variations in cancer management (eg, differential route to diagnosis, staging investigation, treatment) are likely to explain some of the low survival observed in England and among more deprived patients, whereas the role of the individual factors in the observed variations in management seems minor. For example, the background consultation rate in primary care of patients with cancer does not differ between routes to cancer diagnosis (emergency presentation or not).^61^ ^62^ In contrast, interventions on healthcare system factors might have a large impact on cancer survival, as shown by the recent changes in the management of patients with lung cancer.^43^ However, such interventions have not influenced the socioeconomic inequalities in cancer survival yet, possibly because they do not directly address the differential interactions between the healthcare system and the patients, which could lead to suboptimal management of subgroups of the population.

### Conclusion and policy implications

Little evidence has been found about the acceleration in cancer survival after the successive national cancer policy initiatives. Survival in the most deprived has been consistently lower and the deprivation gap has shown little change over the years for patients with a diagnosis during 1971-90^2^ and 1986-99^63^ in England and Wales. This study contributes with more recent data and updates evidence that the deprivation gap persisted in England even after the introduction of successive national policies, which among other goals targeted social inequalities related to cancer.^11^

These findings should be taken into consideration by cancer policy makers and inform future initiatives. Shifting the focus from individual factors to healthcare system factors might prove to be beneficial in improving cancer outcomes among the most disadvantaged. Further research on these factors can help shed light and improve the efficacy of future cancer policies.

**What is already known on this topic**

Cancer survival in England has been improving steadily for all deprivation groups since the 1970s, but still lags behind that seen in comparable countries in Europe
A “deprivation gap” in survival persists between the least and the most deprived in England

**What this study adds**

Even though increasing cancer survival and reducing inequalities in survival have been among the main targets of national cancer policy initiatives implemented since 2000, this study found little evidence of a direct impact of these strategies on one year survival, and no evidence for a reduction in socioeconomic inequalities in survival

## Notes

Ethical approval: This study was approved by the Confidentiality Advisory Group and the Research Ethics Committee (PIAG 1-05(c)/2007 and REC 13/LO/0610). The analysed data were anonymised and none of the results is at individual level and for small groups. Patients are data subjects under the Data Protection Act 1998, but not participants in the research, in the sense that they are not invited to participate because it is observational research for public health surveillance, not interventional research, which certainly would require informed patient consent under law. Furthermore, consent from the patients or their proxies was not sought as it would not be feasible for over 3.5 million cancer patients, many already deceased. The impracticality of seeking consent from such large numbers of patients has been recognised from various bodies that the Cancer Survival Group has sought approvals from such as the Patient Information Advisory Group, the National Information Governance Board and the Confidentiality Advisory Group. All those bodies have acknowledged in the past that obtaining consent is not a viable proposition for population-based research on cancer survival that requires national data from cancer registries. They have always granted permission for this type of research to proceed without consent under Section 251 of the NHS Act 2006 (and previously under Section 60 of the Health and Social Care Act 2001).

Data sharing: This study was based on the English national cancer registry data. The authors do not own these data and hence are not permitted to share them in the original form (only in aggregate form, eg, publications). At the time of request data were provided by the Office for National Statistics but now all cancer registrations are owned and maintained by Public Health England.

Transparency: The lead author (AE) affirms that the manuscript is an honest, accurate, and transparent account of the study being reported; that no important aspects of the study have been omitted; and that any discrepancies from the study as planned have been explained.
